# Understanding the changes in endogenous GA_3_ in relation to developmental transitions in cauliflower (*Brassica oleracea* var. *botrytis* L.)

**DOI:** 10.1371/journal.pone.0321599

**Published:** 2025-06-24

**Authors:** Sohamkumar Luhana, Shrawan Singh, Shivani Nagar, Anamika Chandel, Kishor Varotariya, Manisha Mangal, Aditi Kundu, Navinder Saini, Anil Khar

**Affiliations:** 1 Division of Vegetable Science, ICAR-Indian Agricultural Research Institute, New Delhi, Delhi, India; 2 Division of Plant Physiology, ICAR-Indian Agricultural Research Institute, New Delhi, Delhi, India; 3 Division of Agricultural Chemicals, ICAR-Indian Agricultural Research Institute, New Delhi, Delhi, India; 4 Division of Genetics, ICAR-Indian Agricultural Research Institute, New Delhi, Delhi, India.; NBPGR: National Bureau of Plant Genetic Resources, INDIA

## Abstract

Cauliflower is a crop with intricate developmental transitions influenced by both external and internal cues. Plant growth regulators (PGRs) play a key role in developmental transitions and plant responses to environmental factors. The present study aimed to investigate the endogenous levels of gibberellins (GAs) (i) at six different developmental stages and (ii) at four time points in four varieties of cauliflower using high-performance liquid chromatography (HPLC). The Pusa Ashwini, Pusa Sharad, Pusa Shukti, and Pusa Snowball Kt-25 varieties represent all four thermosensory-based maturity groups: early (20–27 °C), mid-early (15–20 °C), mid-late (12–16 °C), and late or snowball (10–16 °C), respectively. GA_3_ content was highest in Pusa Shukti (4.020 ppm) and lowest in Pusa Ashwini (3.091 ppm). A higher endogenous GA_3_ concentration was recorded at the bolting stage (4.118 ppm), seedling stage (4.057 ppm), and curd initiation stage (3.722 ppm), suggesting its role in stalk elongation. The difference in GA_3_ content was significant between curd (3.613 ppm) and leaf tissues (2.947 ppm) at the full curd stage and nonsignificant between stalk (3.948 ppm) and leaf tissues (4.118 ppm) at the bolting stage. Regarding the time points, the GA_3_ content was highest in Pusa Sharad (4.311 ppm) and lowest in Pusa Ashwini (2.990 ppm). GA_3_ content showed a significant positive correlation with duration to crucial developmental transitions, namely the curd initiation stage, the full curd stage, and the bolting stage. The study highlights the role of endogenous gibberellins in plant development and suggests their potential for benefiting seed production.

## Introduction

For successful reproduction and genetic continuation, it is essential for plants to transition from the vegetative to the reproductive phase and produce flowers and seeds. Floral transition depends on the plant’s response to internal (endogenous) and external (environmental) cues. In the model plant *Arabidopsis*, extensive studies have revealed six major pathways deciding flowering time: photoperiod, vernalization, gibberellins, thermosensory (ambient temperature), age, and autonomous [1]. Gibberellins (GAs) promote flowering by upregulating the expression of floral integrators, such as *LEAFY* (*LFY*) and *SUPPRESSOR OF OVEREXPRESSION OF CO 1* (*SOC1*), via DELLA protein*-*regulated mechanisms [2,3]. GAs govern a number of processes throughout the plant life cycle, including seed germination, stem and leaf growth, trichome development, flowering time, and vegetative and reproductive development [4]. The role of GAs in flowering time regulation has been extensively studied for several years, and it has been proven that flowering defects occur when there is a loss of function in various components of GA biosynthesis and signalling transduction [5]. In contrast, early flowering is induced by exogenous GAs or by elevated endogenous GA levels and enhanced GA signalling [5]. However, the effect of GA being either promoting, inhibiting, or neutral is also dependent on the plant species [6]. Cauliflower (*Brassica oleracea* var. *botrytis* L.) is a key member of the Cole group of vegetable crops and is cultivated in almost 97 countries on an accumulated area of 1.378 million hectares [7]. Cauliflower originated in the Mediterranean region and spread to different parts of the world, including India. Adaptational changes led to the evolution of different ecotypes of cauliflower, such as Originals, Northern, Cornish, Roscoff, Angers, Snowball, and tropical or Indian cauliflower. These evolutionary changes mostly affected the genomic regions associated with developmental transitions [8], which could also be the plant growth regulator (PGR) pathways [6]. Duclos and Björkman (2015) identified prominent GAs in cauliflower as GA_8_, GA_29_, GA_1_, GA_20_, and GA_19_, and in broccoli as GA_8_, GA_29_, GA_1_, and GA_20_ [6]. It is considered that cauliflower directly evolved from broccoli through stepwise domestication involving major gene mutations [9]. The absence of GA_19_, an immediate precursor to GA_20_, in broccoli suggests a possible divergence in the GA biosynthesis pathway between these two *B. oleracea* varieties during the domestication process.

The edible portion of cauliflower, i.e., the curd, is composed of arrested prefloral fleshy inflorescence meristems, marking the vegetative-to-generative transition in two phases. First, the transition from vegetative to inflorescence meristem occurs, followed by elongation of curd, known as bolting, under congenial conditions, leading to the transition from inflorescence meristem to floral primordium. However, these developmental transitions rely on genotypic responses to environmental conditions. Indian cauliflower has four maturity groups that respond to different temperature regimens for developmental transitions [10].

In *Brassica* vegetables, numerous studies have indicated that the application of exogenous GA_3_ induces early bolting and flowering, as well as elongation of stems and stalks [6,11–16], suggesting its potential impact on the endogenous hormone system. It also enhances morphological traits related to vegetative growth, curd, and seed yield in cauliflower [17–19]. The pathway of GA biosynthesis during the production of bioactive end products divides into two branches at GA_12_: one involves hydroxylation at C-13, known as the 13-hydroxylation pathway, resulting in the formation of 13-hydroxylated GAs such as GA_1_, GA_5_, and GA_3_, while the second, i.e., the non-13-hydroxylation pathway, results in the formation of GA_4_ and GA_7_ [20]. Early studies reported endogenous GAs from both pathways in cabbage shoots [21]. In cauliflower, the application of GA_4+7_ (GAs from the non-13-hydroxylation pathway) was shown to promote curd initiation [22,23], while a study analysing endogenous GA_1_ (GA from the 13-hydroxylation pathway) identified it as a possible factor governing inflorescence stalk elongation [11]. However, a recent study in cauliflower reported that endogenous GAs from both the early 13-hydroxylation pathway (specifically, GA_1_, GA_3_, precursors, and inactive metabolites) and the non-13-hydroxylation pathway (GA_4_) are evident in the inflorescence meristems of cauliflower, and exogenous application of both GA_3_ and GA_4+7_ shortens the vegetative period and accelerates the vegetative to reproductive transition [6]. Therefore, in addition to its exogenous applications, GA_3_ also serves endogenously as a crucial factor in the developmental physiology of cauliflower. To date, only a few studies on cauliflower have examined endogenous hormones, primarily focusing on reproductive development [6,11]. A notable gap remains in understanding how the concentration of endogenous GA_3_ varies among the different thermo-sensitive maturity groups and influences different developmental transitions. Therefore, the present study was designed to investigate the natural fluctuations in endogenous GA_3_ concentrations across different developmental transitions and time frames under ambient temperature conditions without any external treatment. To explore the potential role of GA₃ in temperature resilience and its implications for advancing cauliflower improvement for broader production plasticity, we also investigated the association between endogenous GA₃ dynamics and phenological transitions. This study included four maturity groups of cauliflower, namely, early, mid-early, and mid-late groups indigenous to India, as well as the late or snowball group.

## Materials and methods

### Plant material and phenological observations

Four varieties, one from each maturity group: Pusa Ashwini (early), Pusa Sharad (mid-early), Pusa Shukti (mid-late), and Pusa Snowball Kt-25 (PSB Kt-25) (late), were used in the study. The present study included two sowing-time points: (i) for the first experiment (i.e., stage-specific changes in GA content in different thermosensory groups) on 30 August 2022; and (ii) for the second experiment (i.e., changes in GA content at different growth time points) on 30 October 2022. The sowing was done in 12-inch-high earthen pots filled with a mixture of farm soil and vermicompost (2:1 ratio). One-month-old seedlings were subsequently transplanted into raised beds prepared inside the net house (a 40-mesh nylon net). The crop was raised following recommended cultivation practices [24]. Varieties were observed for critical developmental transitions, namely, the young stage (8 leaves) (YS), the adult stage (12 leaves) (AS), the curd initiation (CI) stage, the full curd (FC) stage, and the bolting stage (BS). Stalk length (SL) was measured at the full flowering stage, with an exception for PSB Kt-25, which bolted but did not flower; in this case, the stalk length at the full bolting stage was considered. The daily temperature data of the experimental site during the trial crop period were obtained from the Agro-Met Advisory, Division of Agricultural Physics, ICAR-IARI, New Delhi.

### Determination of GA_3_ content

**Fig 1:**
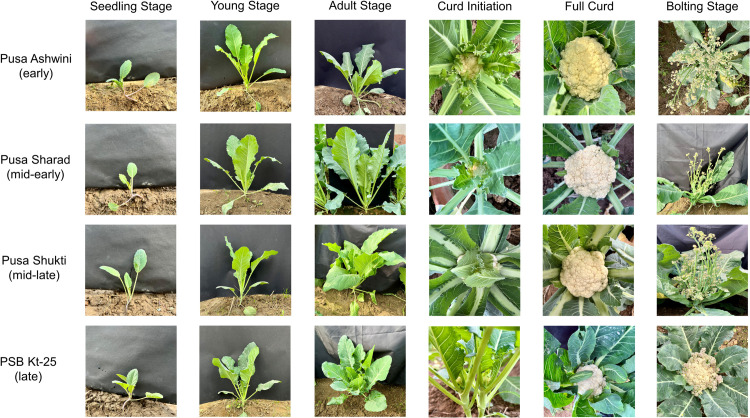
Developmental transitions observed in cauliflower varieties representing four different maturity groups.

To ascertain whether the changes in GA_3_ content are attributed to developmental transitions or temporal factors, the GA_3_ content was determined in two distinct experiments. First, the GA_3_ content was analysed at six crucial developmental transitions: seedling (4 leaves) stage (SS), young stage (YS), adult stage (AS), curd initiation (CI) stage, full curd (FC) stage, and bolting stage (BS) in two replications. For the full curd and bolting stages, in addition to leaf tissues, GA_3_ content was also determined in curd and stalk tissues, respectively. These six developmental transitions in the four varieties are depicted in **Fig 1**. Second, the GA_3_ content in leaf tissues was analysed at four time points: 35, 55, 75, and 95 days after transplanting (DAT), with two replications. Samples were collected from mature leaves in the evening and stored at -80 °C until further processing.

### Preparation of samples for GA_3_ analysis by high-performance liquid chromatography (HPLC)

The GA_3_ samples were prepared following the protocol described by [25] and [26] with minor modifications (S1 Fig). Each 2-gram sample was crushed with liquid nitrogen and homogenized with 10 ml of cold sodium phosphate buffer (0.05 M, pH 7.5) containing 0.02% sodium diethyldithiocarbamate, an antioxidant. After overnight shaking at 4 °C and 150 rpm, the prepared extracts were centrifuged at 10,000 rpm for 10 minutes at 4 °C. The resulting supernatants were collected and adjusted to a final volume of 10 ml with sodium phosphate buffer. The samples were then partitioned using a separating funnel with 5 ml of diethyl ether, and the aqueous phase was collected. The pH of the resulting aqueous phase was adjusted to 2.5 using 1 N HCl. The aqueous phase was partitioned twice with 5 ml of petroleum ether, with each aqueous phase being collected and the ether phase discarded. The aqueous phase was further partitioned three times with 5 ml of diethyl ether. For the first two times, the aqueous phase was collected, and in the last step, the ether phase was taken and filtered through sodium sulphate crystals using Whatman Number 1 filter paper (Whatman plc, UK). The filtrate was collected and left overnight for lyophilization in a small beaker. The next day, the beaker was rinsed with 1 ml of 26% acetonitrile (mobile phase) prepared using HPLC water, filtered through a polyvinylidene difluoride (PVDF) membrane filter (diameter: 13 mm, filtration rate: 0.45 µm), and stored at -20 °C for the determination of GA_3_.

### HPLC protocol

The GA_3_ analysis in the samples was conducted using HPLC (Agilent 1100/1200 series, Agilent Technologies, Santa Clara, CA, USA) at the Division of Plant Physiology, ICAR-IARI, New Delhi. The separation was achieved on a C18 column (Agilent Eclipse XDB-C18, 5 µm pore size, 4.6 × 200 mm; Santa Clara, CA, USA) maintained at 30 °C. The mobile phase consisted of acetonitrile: water (26:74; v/v, pH 3.5) filtered through a PVDF membrane filter. The flow rate was maintained at 0.8 mL/min, and a 20 µL sample of the extract was injected into the column. The wavelength for the determination was 206 nm for UV monitoring and 360 nm for recording. GA_3_ served as an external standard (0.1, 0.5, 1, 5 and 10 mg/L) for identifying the hormone in plant samples. The peak area was utilized to determine the hormone concentration using the formula: GA_3_ concentration (ppm) = peak area/graph factor. The graph factor was obtained from the standard graph (S2 Fig).

### Statistical analysis

Statistical analysis for GA_3_ content data was performed using a two-factor randomized block design (RBD) to determine the interactive effect (I) of genotype (G) and developmental transitions (DT) or plant parts (PP) or time points (TP) on the endogenous GA_3_ concentration and a one-factor RBD for phenological observations by using RStudio packages agricolae and doebioresearch. The normality of the distribution of the data was confirmed with the Shapiro-Wilk normality test. Duncan’s multiple range test (DMRT) at the 5% significance level was used to identify significant differences among treatment means. Replication-wise data is provided in the Supporting Information (S1–S5 Tables). Changes over the seedling stage and 35 DAT were computed using MS Excel. Graphical representation and correlation analysis were accomplished using OriginPro 2023b.

## Results

### Phenological observations

**Fig 2:**
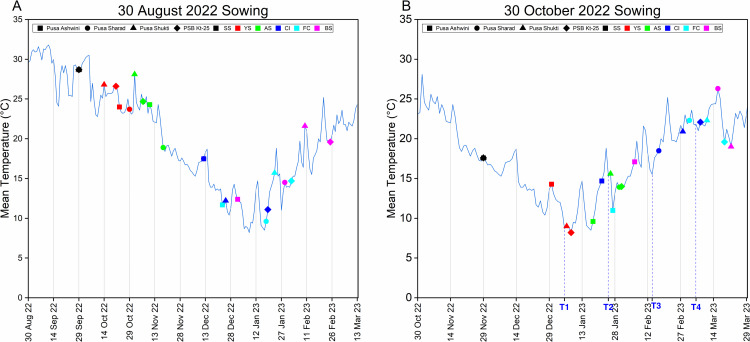
Days to developmental transitions in relation to temperature in four varieties of cauliflower. (A) Developmental transitions for the 30 August 2022 sowing. (B) Developmental transitions and plant developmental stage corresponding to time points for the 30 October 2022 sowing. (Source of temperature data: Division of Agricultural Physics, ICAR-IARI) SS - Seedling Stage, YS - Young Stage, AS - Adult Stage, CI - Curd Initiation, FC - Full Curd, BS - Bolting Stage.

Concerning the 30 August 2022 sowing, Pusa Ashwini (early) and Pusa Sharad (mid-early) were the earliest for the CI stage at 104 days (**Table 1**). Pusa Ashwini progressed quickly, with the FC stage at 115 days and bolting at 124 days, while Pusa Sharad had the CI stage at 141 days and the BS at 152 days. Pusa Shukti (mid-late) and PSB Kt-25 (late) attained the CI stage at 117 and 142 days, respectively. SL was highest in Pusa Shukti (78.67 cm) and lowest in PSB Kt-25 (15.33 cm). For 30 October 2022 sowing, 35 DAT, all genotypes were at the YS (8-leaf stage). Pusa Ashwini reached the FC stage without attaining the proper juvenile or adult stage (AS) by 55 DAT (**Table 2**). By 95 DAT, Pusa Ashwini exhibited flower termination, Pusa Sharad exhibited bolting initiation, and Pusa Shukti and PSB Kt-25 exhibited CI stage. The temperature influence on the developmental transitions of both sowings is depicted in **Fig 2**.

**Table 1:** Days to developmental transitions and stalk length during 30 August 2022 sowing.

Variety	YS	AS	CI	FC	BS	SL (cm)
Pusa Ashwini	54^b^	72^b^	104^c^	115^d^	124^d^	47.33^b^
Pusa Sharad	60^a^	80^a^	104^c^	141^c^	152^c^	49.33^b^
Pusa Shukti	45^c^	63^c^	117^b^	146^b^	164^b^	78.67^a^
PSB Kt-25	52^b^	68^bc^	142^a^	156^a^	179^a^	15.33^c^
Critical Difference (CD) 5%	4.6	6.1	5.6	2.2	4.1	5.9

The lowercase letters represent a significant difference at p<0.05 within the column. YS - Young Stage, AS - Adult Stage, CI - Curd Initiation, FC - Full Curd, BS - Bolting Stage, SL - Stalk Length

**Table 2:** Stages corresponding to time points during 30 October 2022 sowing.

Variety	35 DAT	55 DAT	75 DAT	95 DAT
Pusa Ashwini	YS	FC	FI	FT
Pusa Sharad	YS	AS	CI	Curd Loosening
Pusa Shukti	YS	AS	AS	CI
PSB Kt-25	YS	AS	AS	CI

DAT - Days After Transplanting, YS - Young Stage, FC - Full Curd, FI - Flower Initiation, FT - Flower Termination, AS - Adult Stage, CI - Curd Initiation

### Endogenous GA_3_ content at developmental transitions

Both genotype and developmental transitions, as well as their interactive effect, showed statistically highly significant differences at the 0.1% level (**Table 3**).

**Table 3:** ANOVA for the interactive effect of genotype x developmental transitions/ plant parts/ time points on GA_3_ content of cauliflower.

Source of Variation	DF	Developmental transitions (DT)	DF	Plant parts (PP)			DF	Time points (TP)
				Leaf vs Curd	Leaf vs Stalk	Curd vs Stalk		
Replication	1	0.01	1	0.07	0.02	0.002	1	0.03
Genotype (G)	3	2.5^***^	3	0.7^*^	0.9^**^	0.1	3	2.4^***^
DT/PP/TP	5	1.5^***^	1	1.4^**^	0.1	0.6^*^	3	0.2^**^
Interaction (I)	15	0.6^***^	3	2.2^***^	0.01	1.7^***^	9	0.7^***^
Error	23	0.06	7	0.1	0.06	0.06	15	0.03
CD 5% (G, DT/PP/TP, I)		0.4, 0.4, 0.9		0.6, 0.4, 0.8	0.4, 0.3, 0.6	0.4, 0.2, 0.6		0.3, 0.3, 0.4

*** Significant at 0.1%,

** Significant at 1%,

* Significant at 5%, when tested against MSS due to error. DF - Degrees of Freedom

The endogenous GA_3_ content in the leaves was highest at the bolting stage (4.118 ppm), which was on par with the seedling stage (4.057 ppm) but significantly higher than the GA_3_ content at the full curd stage (2.988 ppm), young stage (3.339 ppm), adult stage (3.507 ppm), and curd initiation stage (3.722 ppm) (**Table 4**). Among the varieties, the maximum GA_3_ content was observed in Pusa Shukti (4.020 ppm), and the minimum was observed in Pusa Ashwini (3.091 ppm). The GA_3_ content in the mid-late group variety Pusa Shukti was comparable to the late group variety PSB Kt-25 (3.994 ppm). The mid-early genotype Pusa Sharad (3.383 ppm) exhibited a higher GA_3_ content than the early group genotype Pusa Ashwini (3.091 ppm). The interactive effect revealed maximum GA_3_ content in PSB Kt-25 at the seedling stage (5.088 ppm), followed by Pusa Shukti at the bolting stage (4.462 ppm) and its adult stage (4.443 ppm).

**Table 4:** Interactive effect of genotype x developmental stages on GA_3_ content (ppm) in leaf portion of cauliflower (sowing date: 30 August 2022).

Genotype	Developmental stages						Mean
	Seedling stage	Young stage	Adult stage	Curd initiation stage	Full curd stage	Bolting stage	
Pusa Ashwini	4.115^bcde^	2.279^k^	2.791^j^	3.339^gh^	1.794^k^	4.227^bcd^	3.091^c^
Pusa Sharad	3.317^ghi^	3.500^fgh^	3.558^fgh^	3.596^fgh^	2.838^ij^	3.490^fgh^	3.383^b^
Pusa Shukti	3.707^efgh^	3.401^gh^	4.443^b^	4.184^bcde^	3.922^cdef^	4.462^b^	4.020^a^
PSB Kt-25	5.088^a^	4.177^bcde^	3.235^hij^	3.771^defg^	3.400^gh^	4.292^bc^	3.994^a^
Mean	4.057^a^	3.339^c^	3.507^bc^	3.722^b^	2.988^d^	4.118^a^	

The lowercase letters in the table represent a significant difference at p<0.05.

**Fig 3:**
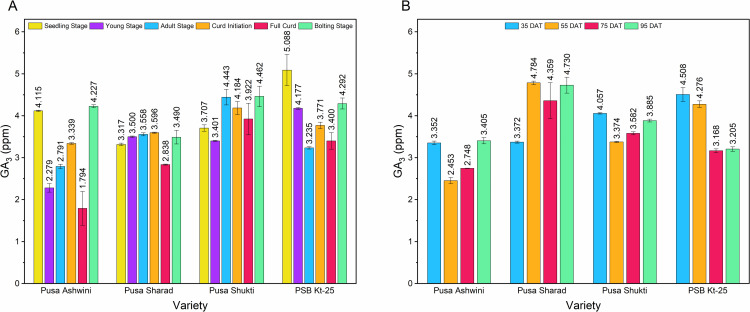
GA_3_ concentration (ppm) in four varieties of cauliflower in two distinct experiments. (A) GA_3_ content (ppm) at developmental transitions. (B) GA_3_ content (ppm) at time points. DAT - Days After Transplanting.

Except for Pusa Sharad, a reduction in the GA_3_ content from the seedling to the young stage was observed for the remaining three varieties (**Fig 3A**). At the adult stage, the GA_3_ content increased in Pusa Ashwini (2.791 ppm) and Pusa Shukti (4.443 ppm), decreased in PSB Kt-25 (3.235 ppm), and remained almost the same in Pusa Sharad (3.558 ppm). During curd initiation, GA_3_ content increased significantly in Pusa Ashwini (3.339 ppm) and in the PSB Kt-25 (3.771 ppm), remained nearly the same in Pusa Sharad (3.596 ppm), and slightly decreased in Pusa Shukti (4.184 ppm). At the full curd stage, the GA_3_ content decreased in all the varieties, with a significant reduction in Pusa Ashwini (1.794 ppm) and Pusa Sharad (2.838 ppm). The bolting stage showed a significant increase in GA_3_ content in all the tested varieties, with the maximum content observed in Pusa Shukti (4.462 ppm), followed by PSB Kt-25 (4.292 ppm), Pusa Ashwini (4.227 ppm), and Pusa Sharad (3.490 ppm).

At the full curd stage, notable differences in GA_3_ content were observed in both the leaf (2.947 ppm) and curd (3.613 ppm) sections, a pattern consistent across varieties except for PSB Kt-25 (late) (**Table 5**). Among these varieties, Pusa Sharad had the highest GA_3_ content (3.729 ppm), followed closely by Pusa Shukti (3.343 ppm), while Pusa Ashwini had the lowest concentration (2.731 ppm). Specifically, within curd tissues, Pusa Sharad (mid-early) had the maximum content (4.620 ppm), while Pusa Shukti had the minimum content (2.765 ppm). Interestingly, at the bolting stage, no significant difference was observed in the leaf (4.118 ppm) and stalk (3.948 ppm) portions (**Table 5**). Within the stalk tissues, Pusa Shukti had the highest GA_3_ content (4.450 ppm), whereas Pusa Sharad displayed the lowest concentration (3.246 ppm). Significant differences were observed in the curd (3.572 ppm) and stalk (3.948 ppm) portions collected from the full curd and bolting stages, respectively. Except for Pusa Ashwini, the tested varieties showed significant differences in GA_3_ content in the curd and stalk portions sampled from their respective stages.

**Table 5:** GA_3_ content (ppm) in leaf and curd, leaf and stalk, and curd and stalk portions of cauliflower varieties.

Genotype	Full curd stage			Bolting stage			Full curd vs bolting stage		
	Leaf	Curd	Mean	Leaf	Stalk	Mean	Curd	Stalk	Mean
Pusa Ashwini	1.794^d^	3.668^b^	2.731^b^	4.227^a^	4.052^ab^	4.140^a^	3.668^bc^	4.052^ab^	3.860^a^
Pusa Sharad	2.838^c^	4.620^a^	3.729^a^	3.490^bc^	3.246^c^	3.368^b^	4.620^a^	3.246 cd	3.933^a^
Pusa Shukti	3.922^ab^	2.765^c^	3.343^a^	4.462^a^	4.450^a^	4.456^a^	2.765^d^	4.450^a^	3.608^a^
PSB Kt-25	3.400^bc^	3.234^bc^	3.317^a^	4.292^a^	4.044^ab^	4.168^a^	3.234 cd	4.044^ab^	3.639^a^
Mean	2.947^b^	3.613^a^		4.118^a^	3.948^a^		3.572^b^	3.948^a^	

The lowercase letters represent a stage-specific significant difference at p<0.05.

Two-factor RBD analysis of variance (ANOVA) for genotypes and plant parts is provided in **Table 3**.

The HPLC chromatograms for one replication are presented in S3 Fig.

### Changes in GA_3_ content over the seedling stage

**Fig 4:**
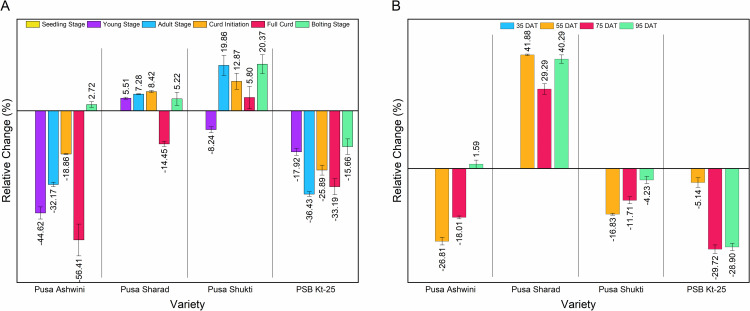
Change (%) in GA_3_ content in four varieties of cauliflower. (A) Change (%) over seedling stage. (B) Change (%) over 35 days after transplanting (DAT).

In the case of Pusa Ashwini, except during the bolting stage, the GA_3_ content consistently remained lower than that in the seedling stage at all the other stages (**Fig 4A**). The most significant reduction, amounting to -56.41%, was noted at the full curd stage, which then showed a slight increase of 2.72% over the seedling stage upon entering the bolting stage. In contrast, except for a decrease of -14.45% at the full curd stage, the GA_3_ content in Pusa Sharad was higher at all the other stages. The highest increase over the seedling stage was observed at the curd initiation stage (8.42%), followed by the adult stage (7.28%). Pusa Shukti maintained a higher GA_3_ content than that in the seedling stage, except during the young stage, where there was a decrease of -8.24%. The most notable increase was observed during the bolting stage (20.37%), followed by the adult stage (19.86%). In PSB Kt-25, the GA_3_ content remained consistently lower than that in the seedling stage at all developmental stages. The most significant reduction was observed in the adult stage (-36.43%), followed by the full curd stage (-33.19%). The bolting stage (-15.66%) and the young stage (-17.92%) showed comparatively lower reductions.

### Endogenous GA_3_ content at four time-points

ANOVA revealed statistically highly significant differences for genotype (p<0.001), time points (p<0.01), and their interactive effect (p<0.001) (**Table 3**).

At 35 DAT, Pusa Ashwini (3.352 ppm) and Pusa Sharad (3.372 ppm) had similar GA_3_ concentrations, while Pusa Shukti (4.057 ppm) and PSB Kt-25 (4.508 ppm) had higher levels (**Table 6**). At 55 DAT, there was a significant decrease in the GA_3_ content in Pusa Ashwini (2.453 ppm) and Pusa Shukti (3.374 ppm), while Pusa Sharad showed a significant increase (4.784 ppm) (**Fig 3B**). PSB Kt-25 exhibited a slight decrease in GA_3_ content (4.276 ppm). At 75 DAT, the GA_3_ content significantly decreased in Pusa Sharad (4.359 ppm) and PSB Kt-25 (3.168 ppm), while it remained relatively constant in Pusa Ashwini (2.748 ppm) and Pusa Shukti (3.582 ppm). At 95 DAT, a significant increase in GA concentration was observed in Pusa Ashwini (3.405 ppm) and Pusa Sharad (4.730 ppm), while Pusa Shukti (3.885 ppm) and PSB Kt-25 (3.205 ppm) had slight increases.

**Table 6:** Interactive effect of genotype x time points on GA_3_ content (ppm) in leaf portion of cauliflower (sowing date: 30 October 2022).

Genotype	35 DAT	55 DAT	75 DAT	95 DAT	Mean
Pusa Ashwini	3.352^fg^	2.453^h^	2.748^h^	3.405^fg^	2.990^c^
Pusa Sharad	3.372^fg^	4.784^a^	4.359^bc^	4.730^a^	4.311^a^
Pusa Shukti	4.057 cd	3.374^fg^	3.582^ef^	3.885^de^	3.724^b^
PSB Kt- 25	4.508^ab^	4.276^bc^	3.168^g^	3.205^g^	3.789^b^
Mean	3.822^a^	3.722^a^	3.464^b^	3.806^a^	

The lowercase letters in the table represent a significant difference at p<0.05. DAT - Days After Transplanting

The HPLC chromatograms for one replication are presented in S3 Fig.

### Changes in GA_3_ content over 35 DAT

In cauliflower, 35-day-old plants correspond to the young stage following which crucial developmental transitions occur; hence, this stage was taken as the base line for calculating the relative change in GA_3_ content. In Pusa Ashwini, the highest reduction (-26.81%) in GA_3_ content compared to 35 DAT occurred at 55 DAT (**Fig 4B**). For Pusa Sharad, the GA_3_ content was higher compared to that at 35 DAT at all time intervals, with the largest increase at 55 DAT (41.88%), followed by 95 DAT (40.29%). Pusa Shukti had a GA_3_ content lower than the base point at all time intervals, with the greatest reduction (-16.83%) at 55 DAT. Like in Pusa Shukti, in PSB Kt-25, the GA_3_ content remained lower than the base point across all time intervals. The largest reduction in PSB Kt-25 was observed at 75 DAT (-29.72%), which was quite close to the value at 95 DAT (-28.90%).

### Correlation analysis

**Fig 5:**
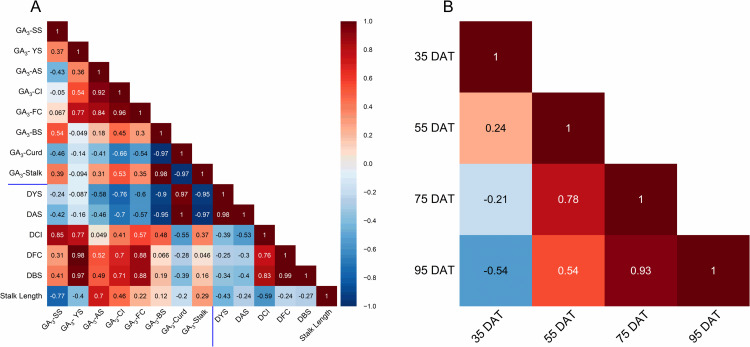
Correlation plots. (A) Correlation plots for GA_3_ content at developmental transitions and phenological observations. (B) Correlation plots for GA_3_ content at time points. SS - Seedling Stage, YS - Young Stage, AS - Adult Stage, CI - Curd Initiation, FC - Full Curd, BS - Bolting Stage, DYS - Days to Young Stage, DAS - Days to Adult Stage, DCI - Days to Curd Initiation, DFC - Days to Full Curd, DBS - Days to Bolting Stage, DAT - Days After Transplanting.

The correlation analysis between the GA_3_ content of developmental transitions and phenological traits is presented in **Fig 5A**. It shows that the GA_3_ content in leaves at full curd stage (FC) was strongly correlated with GA_3_ content in leaves at the young stage (r = 0.77), adult stage (r = 0.84), and curd initiation stage (r = 0.96). However, a negative correlation was observed between the GA_3_ content in the curd portion and the GA_3_ content in the leaf portion from all stages. Furthermore, GA_3_ content at the young stage, adult stage, curd initiation stage, and full curd stage was strongly correlated to days to curd initiation (DCI) stage, days to full curd (DFC) stage, and days to bolting stage (DBS). Similarly, GA_3_ content in leaf and stalk portions at the bolting stage showed a strong positive correlation. Moreover, GA_3_ content at time points at 55 DAT, 75 DAT, and 95 DAT was strongly correlated with each other, pointing to a connection between temporal changes (**Fig 5B**).

## Discussion

Phytohormones encompass a diverse range of chemical compounds that, at micromolar or lower concentrations, play pivotal roles in regulating various aspects of plant growth and development, starting from seed germination and extending to seed maturation and seed dormancy. Gibberellins (GAs) are one of five classical hormones, along with auxins, cytokinins, abscisic acid, and ethylene [27]. Research on gibberellins has advanced for more than a century since their initial discovery [28]. GA_3_ was the first gibberellic acid to be structurally characterized [20] and has been extensively studied for its influence on plant physiology. In contrast to the extensively studied effects of exogenous GA_3_, the changes in endogenous GA_3_ during plant development, especially in cauliflower, are not well understood. To discern the changes in endogenous GA_3_ during plant development, six crucial developmental transitions and four varieties representing each maturity group of cauliflower were selected for the study.

There is cross-talk between abscisic acid (ABA) and GA during germination [29]. GA promotes seed germination, while abscisic acid (ABA) plays a role in initiating and sustaining seed dormancy [30]. Research has shown that both endogenous GAs and the external application of GA_3_ contribute to the promotion of seed germination in many crop plants [31]. External application of GA_3_ was also shown to increase seedling height [31]. Gibberellin biosynthesis in developing seeds causes the accumulation of GA precursors and/or bioactive GAs, such as GA_3_, GA_1_, and GA_4_ [29]. All the tested genotypes had higher concentrations of endogenous GA_3_ at the seedling stage, with a greater concentration in the late-genotype PSB Kt-25 (5.088 ppm), followed by the early cultivar Pusa Ashwini (4.115 ppm), and relatively low concentrations in Pusa Sharad (3.317 ppm) and Pusa Shukti (3.707 ppm). At the molecular level, GA promotes seed germination by enhancing the proteasome-mediated degradation of *RGA-LIKE2* (*RGL2*), a DELLA repressor that inhibits germination, while ABA induces the expression of *ABA-INSENSITIVE5* (*ABI5*), a leucine zipper transcription factor that acts as a repressor of seed germination [32].

In *Arabidopsis*, GAs are known to play a role in the transition from the juvenile to the adult phase, as evident from the increased levels of endogenous GAs during this transition and the induced differentiation of trichomes on the abaxial surface of leaves through GA treatments [33]. A significant increase in internal GA_3_ content was observed in Pusa Ashwini (2.791 ppm) and Pusa Shukti (4.443 ppm) from the young to the adult phase. In contrast, Pusa Sharad (mid-early) exhibited no change, and PSB Kt-25 (late) had a significant reduction in internal GA_3_ concentration from the young to the adult stage. Pertaining to time points, Pusa Sharad had a notable increase in endogenous GA_3_ content from 3.372 ppm at 35 DAT (young stage) to 4.784 ppm at 55 DAT (adult stage).

GAs are renowned for accelerating the vegetative-to-reproductive transition. Endogenous GAs have been shown to be a determinant in the initiation of curd formation in cauliflower [34–36], although the specific GAs involved were not identified. The exogenous application of GA_3_ [6,11,18,37,38] and GA_4+7_ [6,39] has been found to induce earliness in cauliflower and broccoli by reducing the duration of the vegetative phase and the number of vegetative nodes [6]. At the molecular level, however, exogenous GAs did not upregulate the expression of the meristem-identity genes *BoAP1-a* and *BoAP1-c*, which are associated with reproductive transition in cauliflower [6]. In the present study, at the curd initiation stage, all varieties exhibited elevated GA_3_ levels, with Pusa Shukti at 4.184 ppm, PSB Kt-25 at 3.771 ppm, Pusa Sharad at 3.596 ppm, and Pusa Ashwini at 3.339 ppm. Pusa Ashwini and PSB Kt-25 had significant increases in GA_3_ content compared with the adult stage. As far as different time-points are concerned, at 75 DAT, a higher GA_3_ content was observed for Pusa Ashwini (2.748 ppm) and Pusa Sharad (4.359 ppm), while at 95 DAT, Pusa Shukti exhibited increased GA_3_ levels (3.885 ppm). These instances coincided with the curd initiation stage in Pusa Ashwini and near-curd initiation in both Pusa Sharad and Pusa Shukti. The endogenous GA_3_ concentration in leaf tissues decreased significantly when all the genotypes transitioned from the curd initiation stage to the full curd stage. Similar results have been observed with exogenous GAs in previous studies. The application of exogenous GA_3_ and GA_4+7_ during the adult vegetative stage promoted curd initiation, whereas their application to the inflorescence meristem (curd) did not induce further developmental transition [6] or alter the quality of the curd [23]. In broccoli, as well, the exogenous GAs had no effect on the arrested stage, with the developed flower buds remaining unopened [6]. These findings probably suggest the role of other GAs or plant hormones in the post-inflorescence meristem (post-curd) transition.

In the present investigation, a higher concentration of GA_3_ was detected in the curd tissues of the genotypes of all maturity groups, ranging from 2.765 ppm in Pusa Shukti (mid-late) to 4.620 ppm in Pusa Sharad (mid-early), suggesting the significance of GA_3_ in curd development, which was higher than that previously reported [6]. They identified GAs, namely GA_8_, GA_29_, GA_1_, GA_20_, GA_19_, and GA_3_ in the curd tissues of cauliflower through gas chromatography-mass spectrometry (GC-MS), reporting that GA_29_ (0.167 ppm) was the most prominent, followed by GA_19_ (0.004 ppm).

GA plays a pivotal role in promoting plant bolting and flowering [12]. Substantial evidence, including mutant analysis, studies on hormone interactions, and quantitative genetics, strongly supports the role of GAs in stem elongation [40]. GAs are reported as the causal factors for stalk elongation in cabbage and broccoli [6,21]. The application of exogenous GAs has been shown to elongate bolting stalks in cauliflower (GA_3_ [6,11,18], GA_4+7_ [6]), broccoli (GA_3_ [6,16]), cabbage (GA_1_ and GA_4_ [21]), and Chinese cabbage [12]. In cauliflower, a study involving the estimation of endogenous GA_1_ reported that vernalization-mediated increases in GA₁ levels in leaf and stalk tissues play a causal role in stalk elongation [11]. However, in the present study, the endogenous GA_3_ content was significantly higher at the bolting stage in both the leaf and stalk tissues of all four genotypes without vernalization treatment. Furthermore, correlation analysis indicated a strong positive correlation between GA_3_ content in leaf and stalk tissues at the bolting stage, as well as between GA_3_ content in the leaf at the bolting stage and the days to bolting stage. Additionally, stalk length also showed a positive correlation with GA_3_ content at the bolting stage in both leaf and stalk tissues. When analyzed at different time points after transplanting, Pusa Ashwini exhibited a significant increase in endogenous GA_3_ at 95 DAT, coinciding with its bolting stage. These findings suggest that endogenous GA₃ plays a crucial role in regulating the time to bolting and the associated stalk elongation in cauliflower without vernalization.

The GA_3_ content at the full curd stage was strongly correlated with the GA_3_ content at the young stage, adult stage, and curd initiation. Furthermore, a strong positive correlation of the duration to curd initiation, curd maturity, and bolting stage with GA_3_ concentration at the young stage, adult stage, curd initiation, and full curd stage indicates a crucial role of GA_3_ in regulating these developmental transitions [18,22,41]. However, the GA_3_ content at the bolting stage in both leaf and stalk tissues was negatively correlated with the GA_3_ content in curd tissues.

The elevated levels of endogenous GA_3_ during the seedling stage, curd initiation stage, and bolting stage suggest the potential associations of internal GA_3_ with seedling elongation, curd induction, and stalk elongation associated with bolting. The time-point results of GA_3_ content corresponded with the stage of development. To determine the role of phytohormones in developmental transitions, it is recommended that the levels of other endogenous GAs from both the 13-hydroxylation pathway and the non-13-hydroxylation pathway be explored, as it remains unclear whether both pathways are active in cauliflower [6]. Additionally, examining their interactions with other plant hormones is important, as hormonal pathways are interconnected through a complex network of interactions and feedback circuits, which collectively determine the overall effects of individual hormones [42]. To uncover the molecular mechanisms underlying the influence of GAs on developmental stages, expression studies of genes related to the GA pathway and the ambient temperature pathway are also imperative.

## Conclusion

This investigation confirmed the impact of developmental transitions on changes in endogenous GA_3_, indicating the putative role of GA_3_ in governing these transitions. A higher GA_3_ content at the bolting stage suggested an associated role of GA_3_ in stalk elongation during bolting. GA_3_ was also detected at a higher concentration in curd and stalk tissues at the full curd and bolting stages, respectively. Fluctuations at different time points followed the corresponding developmental stages of the plant. The maturity groups exhibited significant variation in GA_3_ content during both developmental transitions and time points.

## Supporting Information

S1 Fig Step-by-step protocol of sample preparation for GA_3_ content analysis by HPLC. (PDF)

S2 Fig Standard curve (Concentration vs Area) of GA_3_. (PDF)

S3 Fig HPLC chromatograms (A) HPLC chromatograms of GA_3_ content at developmental transitions and (B) HPLC chromatograms of GA_3_ content at time points. A peak of GA_3_ was observed at 5.4–5.6.
(PDF)

S1 Table Replication-wise days to developmental transitions and stalk length during 30 August 2022 sowing. (PDF)

S2 Table Replication-wise days to developmental transitions during 30 October 2022 sowing. (PDF)

S3 Table Replication-wise GA_3_ content (ppm) in leaf portion of cauliflower during six developmental transitions (sowing date: 30 August 2022). (PDF)

S4 Table Replication-wise GA_3_ content (ppm) in curd and stalk portions of cauliflower (sowing date: 30 August 2022). (PDF)

S5 Table Replication-wise GA_3_ content (ppm) in leaf portion of cauliflower at four time points (sowing date: 30 October 2022). (PDF)

## References

[1] Cheng J-Z, Zhou Y-P, Lv T-X, Xie C-P, Tian C-E. Research progress on the autonomous flowering time pathway in Arabidopsis. Physiol Mol Biol Plants. 2017;23(3):477–85. doi: 10.1007/s12298-017-0458-3 28878488 PMC5567719

[2] Moon J, Suh S-S, Lee H, Choi K-R, Hong CB, Paek N-C, et al. The SOC1 MADS-box gene integrates vernalization and gibberellin signals for flowering in *Arabidopsis*. Plant J. 2003;35(5):613–23. doi: 10.1046/j.1365-313x.2003.01833.x 12940954

[3] Achard P, Herr A, Baulcombe DC, Harberd NP. Modulation of floral development by a gibberellin-regulated microRNA. Development. 2004;131(14):3357–65. doi: 10.1242/dev.01206 15226253

[4] Jung H, Jo SH, Jung WY, Park HJ, Lee A, Moon JS, et al. Gibberellin promotes bolting and flowering via the floral integrators *RsFT* and *RsSOC1-1* under marginal vernalization in radish. Plants (Basel). 2020;9(5):594. doi: 10.3390/plants9050594 32392867 PMC7284574

[5] Davis SJ. Integrating hormones into the floral-transition pathway of *Arabidopsis thaliana*. Plant Cell Environ. 2009;32(9):1201–10. doi: 10.1111/j.1365-3040.2009.01968.x 19302104

[6] Duclos DV, Björkman T. Gibberellin control of reproductive transitions in *Brassica oleracea* curd development. J Am Soc Hortic Sci. 2015;140(1):57–67. doi: 10.21273/JASHS.140.1.57

[7] FAOSTAT Database. Food and agriculture organisation of the United Nations statistical database; Statistical Division; FAO: Rome, Italy; 2021 [cited 2024 Jan 5] Available from: http://www.fao.org/statistics/en/

[8] Rakshita KN, Singh S, Verma VK, Sharma BB, Saini N, Iquebal MA, et al. Agro-morphological and molecular diversity in different maturity groups of Indian cauliflower (*Brassica oleracea* var. *botrytis* L.). PLoS One. 2021;16(12):e0260246. doi: 10.1371/journal.pone.0260246 34890399 PMC8664203

[9] Chen R, Chen K, Yao X, Zhang X, Yang Y, Su X, et al. Genomic analyses reveal the stepwise domestication and genetic mechanism of curd biogenesis in cauliflower. Nat Genet. 2024;56(6):1235–1244. doi: 10.1038/s41588-024-01744-4 38714866 PMC11176064

[10] Swarup V, Chatterjee SS. Origin and genetic improvement of Indian cauliflower. Econ Bot. 1972;26: 381–393. doi: 10.1007/BF02860710

[11] Guo D-P, Ali Shah G, Zeng G-W, Zheng S-J. The interaction of plant growth regulators and vernalization on the growth and flowering of cauliflower (*Brassica oleracea* var. *botrytis*). Plant Growth Regul. 2004;43:163–71. doi: 10.1023/B:GROW.0000040114.84122.11

[12] Song S, Lei Y, Huang X, Su W, Chen R, Hao Y. Crosstalk of cold and gibberellin effects on bolting and flowering in flowering Chinese cabbage. J Integr Agric. 2019;18(5):992–1000. doi: 10.1016/S2095-3119(18)62063-5

[13] Kou E, Huang X, Zhu Y, Su W, Liu H, Sun G, et al. Crosstalk between auxin and gibberellin during stalk elongation in flowering Chinese cabbage. Sci Rep. 2021;11(1):3976. doi: 10.1038/s41598-021-83519-z 33597591 PMC7889655

[14] Ou X, Wang Y, Zhang J, Xie Z, He B, Jiang Z, et al. Identification of *BcARR* genes and CTK effects on stalk development of flowering Chinese cabbage. Int J Mol Sci. 2022;23(13):7412. doi: 10.3390/ijms23137412 35806416 PMC9266762

[15] Wang Y, Huang X, Huang X, Su W, Hao Y, Liu H, et al. BcSOC1 promotes bolting and stem elongation in flowering Chinese cabbage. Int J Mol Sci. 2022;23(7):3459. doi: 10.3390/ijms23073459 35408819 PMC8998877

[16] Nakajima KM, Ohishi M, Sato F, Takahashi M. Gibberellin-induced stem elongation and apical bud growth acceleration without decreased yield in broccoli (*Brassica oleracea* L. var. *italica*). Hortic J. 2023;92(3): 281–9. doi: 10.2503/hortj.QH-045

[17] Gadhiya D, Champaneri DD, Patel NK. The implications of growth-regulating substances on growth and yield traits of cauliflower. Int J Environ Clim Change. 2023;13(10):1876–81. doi: 10.9734/ijecc/2023/v13i102843

[18] Prodhan MM, Sarker U, Hoque MA, Biswas MS, Ercisli S, Assouguem A, et al. Foliar application of GA_3_ stimulates seed production in cauliflower. Agronomy. 2022;12(6):1394. doi: 10.3390/agronomy12061394

[19] Rahman M, Imran M, Ikrum M. Effects of planting date and growth hormone on growth and yield of cauliflower. J Environ Sci Nat Resourc. 2016;9(2):143–150. doi: 10.3329/jesnr.v9i2.32185

[20] Hedden P. The current status of research on gibberellin biosynthesis. Plant Cell Physiol. 2020;61(11): 1832–1849. doi: 10.1093/pcp/pcaa092 32652020 PMC7758035

[21] Hamano M, Yamato Y, Yamazaki H, Miura H. Endogenous gibberellins and their effects on flowering and stem elongation in cabbage (*Brassica oleracea* var. *capitata*). J Hortic Sci Biotech. 2002;77(2):220–225. doi: 10.1080/14620316.2002.11511483

[22] Booij R. Effect of growth regulators on curd diameter of cauliflower. Sci Hortic. 1989;38(1):23–32. doi: 10.1016/0304-4238(89)90016-2

[23] Booij R. Effects of gibberellic acids on time of maturity and on yield and quality of cauliflower. Neth J Agr Sci. 1990;38(4):641–51. doi: 10.18174/njas.v38i4.16554

[24] Sharma SR, Singh R. Cauliflower. In: Thamburaj S, Singh N, editors. Textbook of Vegetables, Tubercrops and Spices. Indian Council of Agricultural Research: New Delhi; 2003. pp. 76–97.

[25] Kim SK, Son T, Park SY, Lee I, Lee B, Kim H, et al. Influences of gibberellin and auxin on endogenous plant hormone and starch mobilization during rice seed germination under salt stress. J Environ Biol. 2006;27(2):181–6.

[26] Nagar S, Singh VP, Singh N, Dhakar R, Arora A. Effect of endogenous gibberellic acid content on physiological and yield related traits in late sown wheat. Indian J Agric Sci. 2022;92(6):700–704. doi: 10.56093/ijas.v92i6.102508

[27] Kende H, Zeevaart J. The five “classical” plant hormones. Plant Cell. 1997;9(7):1197–210. doi: 10.1105/tpc.9.7.1197 12237383 PMC156991

[28] Hedden P, Sponsel V. A Century of gibberellin research. J Plant Growth Regul. 2015;34(4):740–60. doi: 10.1007/s00344-015-9546-1 26523085 PMC4622167

[29] Villedieu-Percheron E, Lachia M, Jung PM, Screpanti C, Fonné-Pfister R, Wendeborn S, et al. Chemicals inducing seed germination and early seedling development. Chimia (Aarau). 2014;68(9):654–63. doi: 10.2533/chimia.2014.654 25437787

[30] Debeaujon I, Koornneef M. Gibberellin requirement for *Arabidopsis* seed germination is determined both by testa characteristics and embryonic abscisic acid. Plant Physiol. 2000;122(2):415–24. doi: 10.1104/pp.122.2.415 10677434 PMC58878

[31] Shah SH, Islam S, Mohammad F, Siddiqui MH. Gibberellic acid: a versatile regulator of plant growth, development and stress responses. J Plant Growth Regul. 2023;42(12):7352–73. doi: 10.1007/s00344-023-11035-7

[32] Piskurewicz U, Jikumaru Y, Kinoshita N, Nambara E, Kamiya Y, Lopez-Molina L. The gibberellic acid signaling repressor *RGL2* inhibits *Arabidopsis* seed germination by stimulating abscisic acid synthesis and *ABI5* activity. Plant Cell. 2008;20(10):2729–45. doi: 10.1105/tpc.108.061515 18941053 PMC2590721

[33] Barbosa NCS, Dornelas MC. The roles of gibberellins and cytokinins in plant phase transitions. Tropical Plant Biol. 2021;14(1): 11–21. doi: 10.1007/s12042-020-09272-1

[34] Kato T On the flower head formation and development of cauliflower plants. II. Physiological studies on the flower head formation. J Jpn Soc Hortic Sci. 1965;34(1):49–56.

[35] Thomas TH, Lester JN, Salter PJ. Hormonal changes in the stem apex of the cauliflower plant in relation to curd development. J Hortic Sci. 1972;47(4):449–55. doi: 10.1080/00221589.1972.11514488

[36] Wurr DCE, Akehurst JM, Thomas TH. A hypothesis to explain the relationship between low-temperature treatment, gibberellin activity, curd initiation and maturity of cauliflower. Sci Hortic. 1981;15(4):321–30. doi: 10.1016/0304-4238(81)90086-8

[37] Aditya DK, Fordham R. Effects of cold treatment and of gibberellic acid on flowering of cauliflower. J Hortic Sci. 1995;70(4):577–85. doi: 10.1080/14620316.1995.11515330

[38] Cung T. Effects of gibberellic acid and benzyl adenine on growth and yield of broccoli (*Brassica oleracea* var. *italica*). M.Sc. Thesis, Yezin Agricultural University. 2015. Available from: https://meral.edu.mm/record/180/file_preview/tuan%20cung%20MS%20thesis%202015.pdf?allow_aggs=True

[39] Fernández JA, Bañón S, Franco JA, González A, Martínez PF. Effects of vernalization and exogenous gibberellins on curd induction and carbohydrate levels in the apex of cauliflower (*Brassica oleracea* var. *botrytis*). Sci Hortic. 1997;70(2):223–30. doi: 10.1016/S0304-4238(97)00059-9

[40] Wang Y, Zhao J, Lu W, Deng D. Gibberellin in plant height control: old player, new story. Plant Cell Rep. 2017;36(3):391–8. doi: 10.1007/s00299-017-2104-5 28160061

[41] Yacoub S, Abbas H. Effect of gibberellic acid on earliness of cauliflower curd initiation under Assuit conditions. Assiut J Agric Sci. 2012;43(1):45–54. doi: 10.21608/ajas.2012.265991

[42] Vanstraelen M, Benková E. Hormonal interactions in the regulation of plant development. Annu Rev Cell Dev Biol. 2012;28(1):463–87. doi: 10.1146/annurev-cellbio-101011-155741 22856461

