# Supplementary figures and images for: Understanding the changes in endogenous GA3 in relation to developmental transitions in cauliflower (Brassica oleracea var. botrytis L.)
Source: PLoS One. 2025 Jun 24;20(6):e0321599. doi: 10.1371/journal.pone.0321599 (PMC12186969; doi:10.1371/journal.pone.0321599)

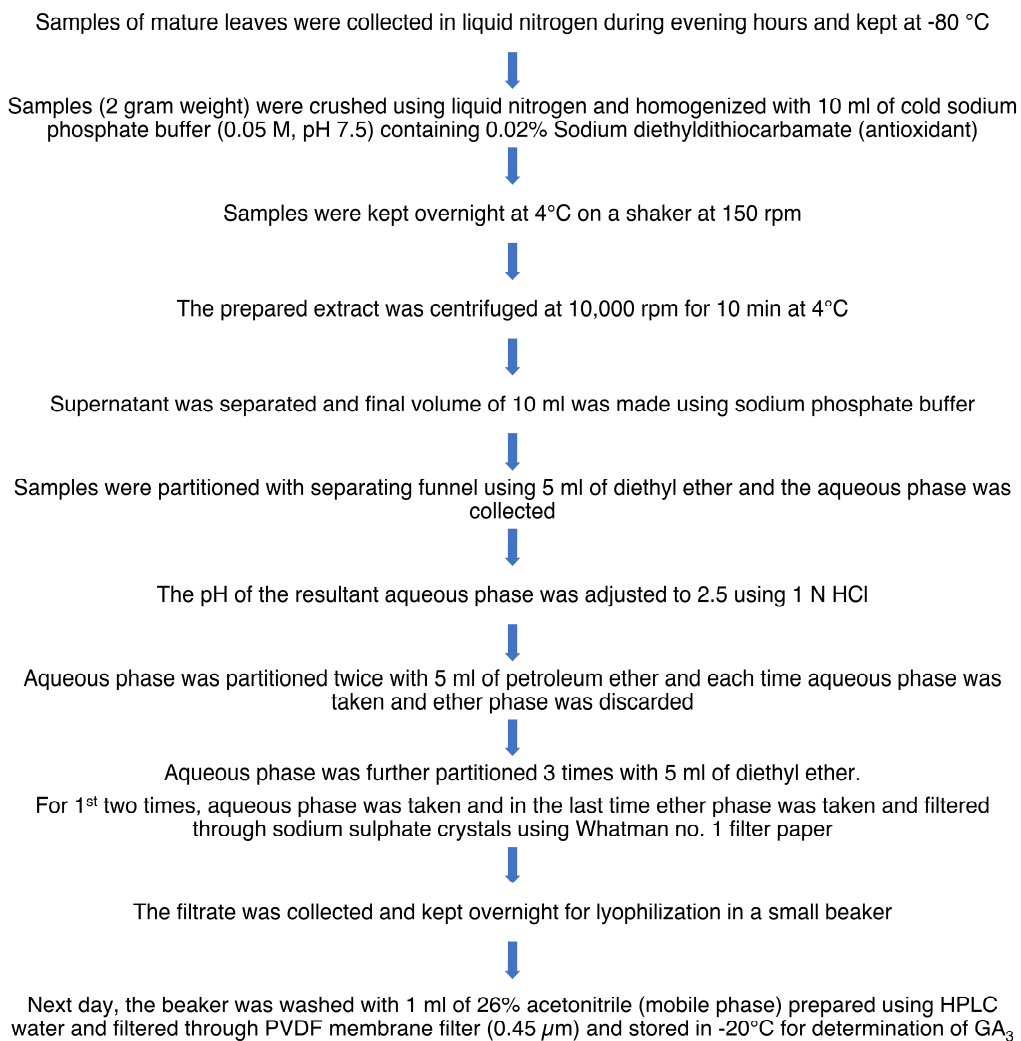

**S1 Fig.** Step-by-step protocol of sample preparation for GA<sub>3</sub> content analysis by HPLC.

Supplement: S1 Fig — (PDF) [file pone.0321599.s001.pdf]

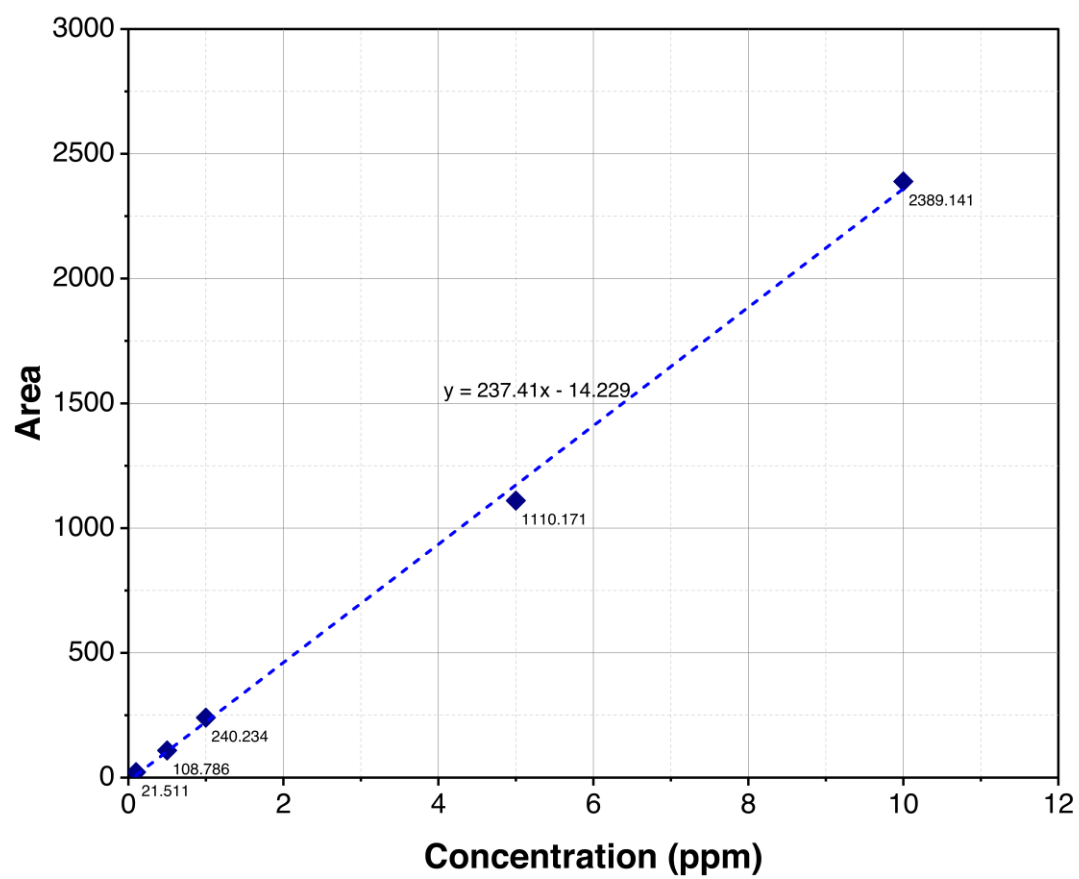

**S2 Fig.** Standard curve (Concentration vs Area) of GA<sub>3</sub>.

Supplement: S2 Fig — (PDF) [file pone.0321599.s002.pdf]
