# Supplementary material for: Understanding the changes in endogenous GA3 in relation to developmental transitions in cauliflower (Brassica oleracea var. botrytis L.)
Source: PLoS One. 2025 Jun 24;20(6):e0321599. doi: 10.1371/journal.pone.0321599 (PMC12186969; doi:10.1371/journal.pone.0321599)
Supplement: S3 Fig — A peak of GA3 was observed at 5.4–5.6. (PDF) [file pone.0321599.s003.pdf]

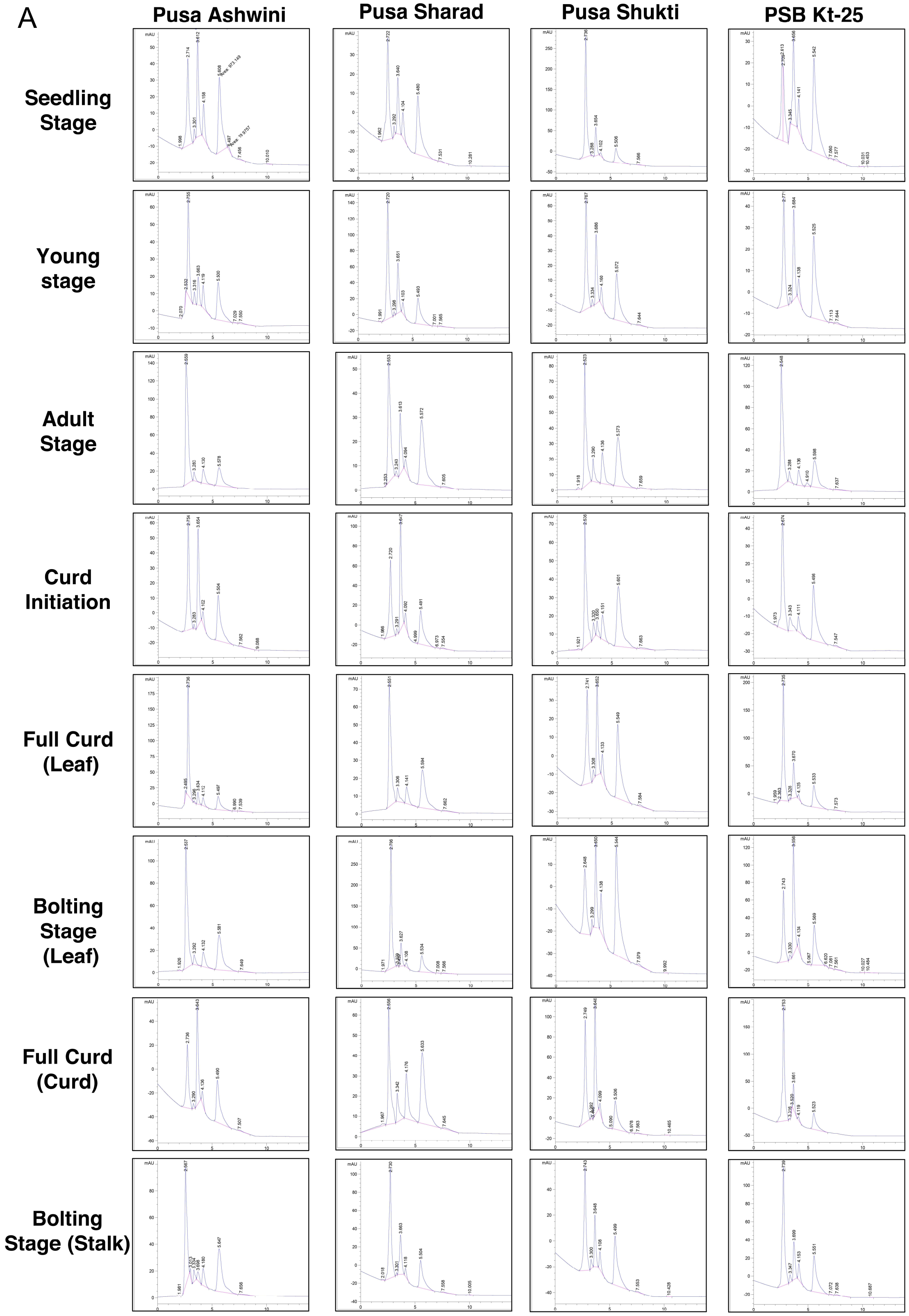
A
Pusa Ashwini
Pusa Sharad
Pusa Shukti
PSB Kt-25
Seedling Stage
Young stage
Adult Stage
Curd Initiation
Full Curd (Leaf)
Bolting Stage (Leaf)
Full Curd (Curd)
Bolting Stage (Stalk)

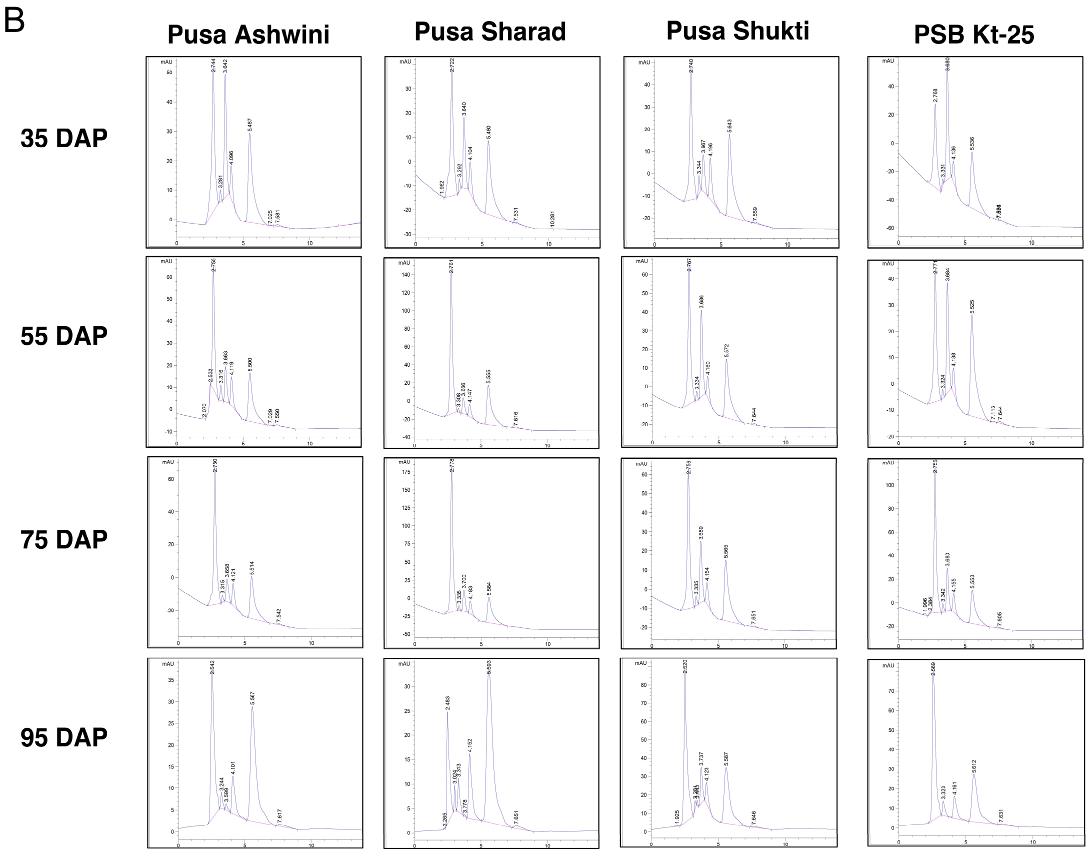

S3 Fig. HPLC chromatograms (A) HPLC chromatograms of GA_3 content at developmental transitions and (B) HPLC chromatograms of GA_3 content at time points. A peak of GA_3 was observed at 5.4-5.6.
