# Supplementary material for: Understanding the changes in endogenous GA3 in relation to developmental transitions in cauliflower (Brassica oleracea var. botrytis L.)
Source: PLoS One. 2025 Jun 24;20(6):e0321599. doi: 10.1371/journal.pone.0321599 (PMC12186969; doi:10.1371/journal.pone.0321599)
Supplement: S1 Table — (PDF) [file pone.0321599.s004.pdf]

S1 Table. Replication-wise days to developmental transitions and stalk length during 30 August 2022 sowing.

Genotype	Days to developmental transitions	Replication 1	Replication 2	Replication 3
Pusa Ashwini	Young stage	52	56	53
	Adult stage	70	70	75
	Curd initiation stage	102	105	105
	Full curd stage	112	116	116
	Bolting stage	123	123	125
	Stalk length (cm)	45	47	50
Pusa Sharad	Young stage	64	60	57
	Adult stage	80	80	80
	Curd initiation stage	105	102	105
	Full curd stage	140	140	144
	Bolting stage	153	150	152
	Stalk length (cm)	50	46	52
Pusa Shukti	Young stage	47	47	42
	Adult stage	65	65	60
	Curd initiation stage	116	119	116
	Full curd stage	146	146	146
	Bolting stage	164	166	162
	Stalk length (cm)	82	74	80
PSB Kt-25	Young stage	54	51	52
	Adult stage	65	70	70
	Curd initiation stage	142	137	146
	Full curd stage	155	155	159
	Bolting stage	180	176	181
	Stalk length (cm)	17	16	13
