# Supplementary material for: Understanding the changes in endogenous GA3 in relation to developmental transitions in cauliflower (Brassica oleracea var. botrytis L.)
Source: PLoS One. 2025 Jun 24;20(6):e0321599. doi: 10.1371/journal.pone.0321599 (PMC12186969; doi:10.1371/journal.pone.0321599)
Supplement: S2 Table — (PDF) [file pone.0321599.s005.pdf]

S2 Table. Replication-wise days to developmental transitions during 30 October 2022 sowing.

Genotype	Days to developmental transitions	Replication 1	Replication 2	Replication 3
Pusa Ashwini	Young stage	46	48	46
	Adult stage	60	60	60
	Curd initiation stage	71	65	68
	Full curd stage	87	91	87
	Bolting stage	92	96	96
Pusa Sharad	Young stage	51	51	54
	Adult stage	71	71	71
	Curd initiation stage	85	87	83
	Full curd stage	115	112	108
	Bolting stage	132	127	124
Pusa Shukti	Young stage	60	52	56
	Adult stage	76	76	76
	Curd initiation stage	108	110	113
	Full curd stage	131	135	135
	Bolting stage	144	147	150
PSB Kt-25	Young stage	57	52	55
	Adult stage	76	76	76
	Curd initiation stage	120	123	120
	Full curd stage	149	142	142
	Bolting stage	157	153	161
