# Supplementary material for: Understanding the changes in endogenous GA3 in relation to developmental transitions in cauliflower (Brassica oleracea var. botrytis L.)
Source: PLoS One. 2025 Jun 24;20(6):e0321599. doi: 10.1371/journal.pone.0321599 (PMC12186969; doi:10.1371/journal.pone.0321599)
Supplement: S3 Table — (PDF) [file pone.0321599.s006.pdf]

S3 Table. Replication-wise GA_3 content (ppm) in leaf portion of cauliflower during six developmental transitions (sowing date: 30 August 2022).

Genotype	Developmental stage	Replication 1	Replication 2	Mean
Pusa Ashwini	Seedling stage	4.099	4.132	4.115
	Young stage	2.379	2.179	2.279
	Adult stage	2.739	2.843	2.791
	Curd initiation stage	3.318	3.360	3.339
	Full curd stage	2.196	1.391	1.794
	Bolting stage	4.263	4.192	4.227
Pusa Sharad	Seedling stage	3.346	3.288	3.317
	Young stage	3.515	3.484	3.500
	Adult stage	3.595	3.522	3.558
	Curd initiation stage	3.611	3.581	3.596
	Full curd stage	2.825	2.850	2.838
	Bolting stage	3.326	3.654	3.490
Pusa Shukti	Seedling stage	3.631	3.782	3.707
	Young stage	3.387	3.416	3.401
	Adult stage	4.627	4.259	4.443
	Curd initiation stage	4.340	4.028	4.184
	Full curd stage	4.299	3.545	3.922
	Bolting stage	4.217	4.706	4.462
PSB Kt-25	Seedling stage	4.715	5.461	5.088
	Young stage	4.198	4.155	4.177
	Adult stage	3.207	3.263	3.235
	Curd initiation stage	3.849	3.693	3.771
	Full curd stage	3.202	3.597	3.400
	Bolting stage	4.421	4.162	4.292
