# Supplementary material for: Understanding the changes in endogenous GA3 in relation to developmental transitions in cauliflower (Brassica oleracea var. botrytis L.)
Source: PLoS One. 2025 Jun 24;20(6):e0321599. doi: 10.1371/journal.pone.0321599 (PMC12186969; doi:10.1371/journal.pone.0321599)
Supplement: S4 Table — (PDF) [file pone.0321599.s007.pdf]

S4 Table. Replication-wise GA_3 content (ppm) in curd and stalk portions of cauliflower (sowing date: 30 August 2022).

Genotype	Plant part	Replication 1	Replication 2	Mean
Pusa Ashwini	Curd	3.692	3.644	3.668
	Stalk	4.089	4.016	4.052
Pusa Sharad	Curd	4.637	4.604	4.620
	Stalk	3.157	3.335	3.246
Pusa Shukti	Curd	2.917	2.612	2.765
	Stalk	4.215	4.684	4.450
PSB Kt-25	Curd	3.009	3.459	3.234
	Stalk	4.278	3.809	4.044
