# Supplementary material for: Understanding the changes in endogenous GA3 in relation to developmental transitions in cauliflower (Brassica oleracea var. botrytis L.)
Source: PLoS One. 2025 Jun 24;20(6):e0321599. doi: 10.1371/journal.pone.0321599 (PMC12186969; doi:10.1371/journal.pone.0321599)
Supplement: S5 Table — (PDF) [file pone.0321599.s008.pdf]

S5 Table. Replication-wise GA_3 content (ppm) in leaf portion of cauliflower at four time points (sowing date: 30 October 2022).

Genotype	Time point	Replication 1	Replication 2	Mean
Pusa Ashwini	35 DAT	3.394	3.310	3.352
	55 DAT	2.379	2.528	2.453
	75 DAT	2.745	2.751	2.748
	95 DAT	3.330	3.480	3.405
Pusa Sharad	35 DAT	3.346	3.398	3.372
	55 DAT	4.740	4.827	4.784
	75 DAT	3.932	4.786	4.359
	95 DAT	4.540	4.920	4.730
Pusa Shukti	35 DAT	4.075	4.039	4.057
	55 DAT	3.387	3.360	3.374
	75 DAT	3.546	3.618	3.582
	95 DAT	3.850	3.920	3.885
PSB Kt-25	35 DAT	4.344	4.671	4.508
	55 DAT	4.198	4.353	4.276
	75 DAT	3.124	3.212	3.168
	95 DAT	3.150	3.260	3.205

DAT- Days After Transplanting
